# Exploring Thiazolopyridine AV25R: Unraveling of Biological Activities, Selective Anti-Cancer Properties and In Silico Target and Binding Prediction in Hematological Neoplasms

**DOI:** 10.3390/molecules28248120

**Published:** 2023-12-15

**Authors:** Annika Ladwig, Shailendra Gupta, Peter Ehlers, Anett Sekora, Moosheer Alammar, Dirk Koczan, Olaf Wolkenhauer, Christian Junghanss, Peter Langer, Hugo Murua Escobar

**Affiliations:** 1 Department of Medicine, Clinic III—Hematology, Oncology and Palliative Care, Rostock University Medical Center, 18057 Rostock, Germany; annika.ladwig@med.uni-rostock.de (A.L.); anett.sekora@med.uni-rostock.de (A.S.); moosheer.alammar@med.uni-rostock.de (M.A.); christian.junghanss@med.uni-rostock.de (C.J.); 2 Department of Systems Biology and Bioinformatics, University of Rostock, 18057 Rostock, Germany; shailendra.gupta@uni-rostock.de (S.G.); olaf.wolkenhauer@uni-rostock.de (O.W.); 3 Institute of Chemistry, University of Rostock, 18057 Rostock, Germany; peter.ehlers@uni-rostock.de (P.E.); peter.langer@uni-rostock.de (P.L.); 4 Core Facility Genomics, Rostock University Medical Center, 18057 Rostock, Germany; dirk.koczan@med.uni-rostock.de

**Keywords:** anti-proliferative, apoptosis, lymphoma, B-ALL, thiazolopyridine, target-screening, molecular docking

## Abstract

Thiazolopyridines are a highly relevant class of small molecules, which have previously shown a wide range of biological activities. Besides their anti-tubercular, anti-microbial and anti-viral activities, they also show anti-cancerogenic properties, and play a role as inhibitors of cancer-related proteins. Herein, the biological effects of the thiazolopyridine AV25R, a novel small molecule with unknown biological effects, were characterized. Screening of a set of lymphoma (SUP-T1, SU-DHL-4) and B- acute leukemia cell lines (RS4;11, SEM) revealed highly selective effects of AV25R. The selective anti-proliferative and metabolism-modulating effects were observed in vitro for the B-ALL cell line RS4;11. Further, we were able to detect severe morphological changes and the induction of apoptosis. Gene expression analysis identified a large number of differentially expressed genes after AV25R exposure and significant differentially regulated cancer-related signaling pathways, such as VEGFA-VEGFR2 signaling and the EGF/EGFR pathway. Structure-based pharmacophore screening approaches using in silico modeling identified potential biological AV25R targets. Our results indicate that AV25R binds with several proteins known to regulate cell proliferation and tumor progression, such as FECH, MAP11, EGFR, TGFBR1 and MDM2. The molecular docking analyses indicates that AV25R has a higher binding affinity compared to many of the experimentally validated small molecule inhibitors of these targets. Thus, here we present in vitro and in silico analyses which characterize, for the first time, the molecular acting mechanism of AV25R, including cellular and molecular biologic effects. Additionally, this predicted the target binding of the molecule, revealing a high affinity to cancer-related proteins and, thus, classified AVR25 for targeted intervention approaches.

## 1. Introduction

Small molecules, defined by a size of <500 DA, can translocate through the cell membrane to interact with intracellular targets and interfere with molecular pathways [1]. This molecule class represents an alternative or supplement to the currently utilized therapeutic options of cancer therapy, surgery, systemic chemotherapy and radiation [2,3]. However, conventional chemotherapy and radiation, in particular, can cause systemic damage to nearby areas or organs [4]. Cytotoxic chemotherapeutics interfere with fast-dividing cells, independent of their adverse effects on neoplastic as well as non-neoplastic cells. Accordingly, the cytotoxic effects impact various organs and compartments, such as bone marrow or hair cells, resulting in the known side effects of chemotherapy [5]. In the case of conventional cancer therapies, the molecular background of the individual patient often does not play a decisive role, in contrast to personalized medicine approaches. Lately, diagnostic Next Generation Sequencing (NGS) characterizing the individual genetic background of each patient and their respective tumors has been introduced, promoting molecular stratified therapeutic treatment [6,7]. These targeted approaches allow us to overcome several challenges of conventional therapies, directly addressing individual present aberrant targets [8,9]. At the same time, the rate of non-responding patients to conventional “one fits all” approaches are reduced, directly affecting the quality of life of the patients. However, despite the striking effects of targeted therapies in selected cancer types, specific side effects are also reported, including delayed fatal effects, as reported for the PI3K inhibitor Idelalisib [10,11]. Further molecular-based therapeutic decisions are currently not routinely used in all cancer entities, as in many cases, specific clinically approved small molecules addressing the genetic landscape are not available. Therefore, in recent years, research has become increasingly focused on developing small molecules that interact with specific targets based on individual molecular landscapes, to reduce systemic side effects [12]. Accordingly, different classes of relevant chemical molecules, including naturally occurring and synthetic substances, were identified as small molecules with anti-cancer activity [13,14]. 

In the medical context, the class of thiazolopyridines represents a class of relevant and noteworthy chemical derivatives. Thiazolopyridine derivatives show various biological activities such as anti-tubercular [15,16], anti-inflammatory [17], anti-microbial [18,19], anti-coagulating [20], anti-viral [21,22] and anti-carcinogenic activity [23,24]. Hence, thiazolopyridines have been unveiled, such as epidermal growth factor receptor (EGFR) tyrosine kinase inhibitors [25], inhibitors of transcriptional regulator (STAT3) [26], mitotic checkpoint inhibitors [27], Interleukin-1 receptor-associated kinases (IRAK4) [28,29,30], Bcl-xL inhibitors [31] and deubiquitinating enzymes (DUBs) [32].

**Figure 1:**
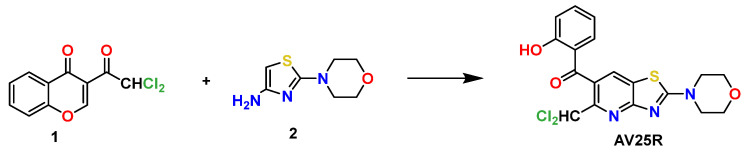
Synthesis of AV25R from Chromone 1 and Enamine 2.

In the present work, we evaluated the biological activity and anti-cancer properties of thiazolopyridine AV25R (5-(dichloromethyl)-2-morpholinothiazolo[4,5-*b*]pyridin-6-yl)(2-hydroxyphenyl)methanone), previously synthesized by the authors [33]. AV25R was selectively synthesized from the reaction of readily available 3-(Dichloroacetyl)chromone 1 with the respective enamine 2 (Figure 1), as previously described, and its chemical structure shows key similarities to known bioactive molecules from this class of substances and its biological activity. In particular, structurally related benzothiazolopyridines and benzooxazolopyridines functionalized with morpholine entities have been reported as potent inhibitors of IRAK4 and phosphoinostidide 3-kinase (PI3K) inhibitors, which are both important targets for anti-cancer therapy, with IC50 values in the nanomolar range [34,35]. However, due to its structural similarities to these known bioactive substances, we investigated the biological activity and anti-cancer properties of AV25, which were previously unknown.

## 2. Results

### 2.1. Cell Biologic Characterisation of AVR25-Induced Effects

#### 2.1.1. Hemolytic Activity

**Figure 2:**
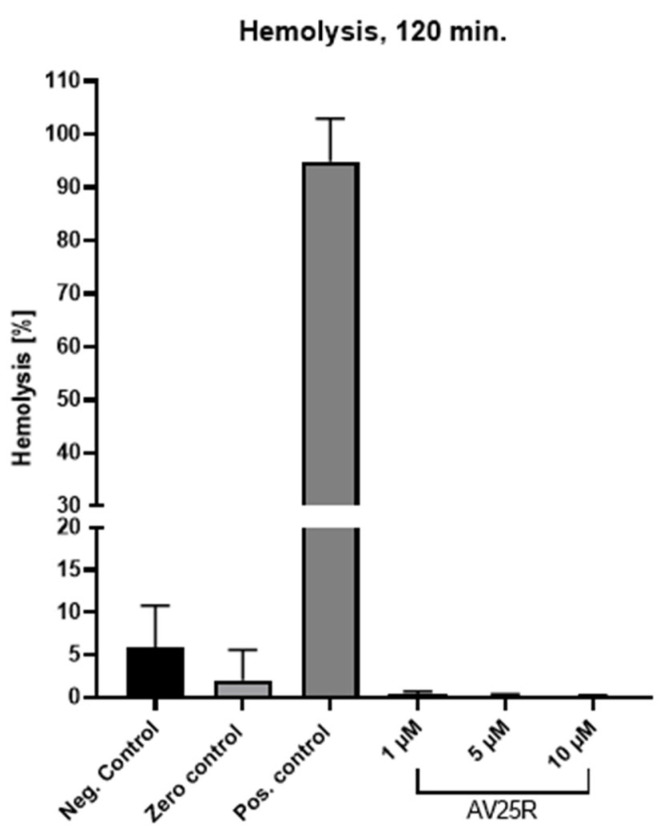
Hemolytic activity of thiazolopyridine AV25R after an incubation period of 2 h. Shown are means ± SD (*n* = 3).

In order to exclude a hemolytic activity of AV25R, its hemolytic potential was evaluated with human whole blood samples from three donors. Concentrations between 1 and 10 µM were evaluated. A non-significant increase in hemolysis was observed after the compound’s application compared to the negative control (only whole blood) or with zero control (whole blood + PBS), with an absence of erythrocyte toxicity (Figure 2).

#### 2.1.2. Evaluation of Proliferation and Metabolic Activity

**Figure 3:**
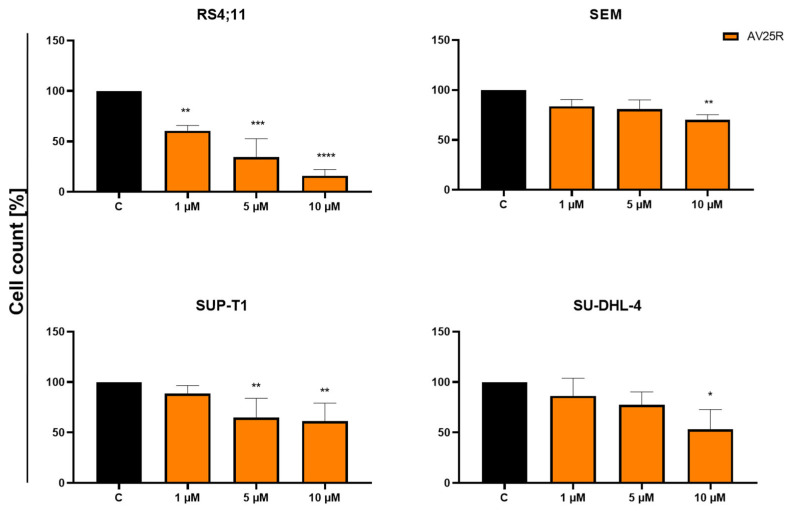
Results of cell count after 48 h of concentration-dependent AV25R exposure on B-ALL cell lines RS4;11 and SEM, as well as lymphoma cell lines SUP-T1 and SU-DHL-4. Data are presented as relative values (DMSO control © was set as 100%) mean ± SD (*n* ≥ 3). Significances were displayed as follows: *: *p* < 0.033, **: *p* < 0.002, ***: *p* < 0.001, ****: *p* < 0.0001.

To evaluate the initial biological response of four human cancer cell lines including two B-ALL cell lines (SEM and RS4;11) and two lymphoma cell lines (SU-DHL-4 and SUP-T1), we analyzed the metabolic activity and proliferation after 48 and 72 h, respectively. AV25R exposure led to a significant reduction in cell proliferation of the B-ALL cell line RS4;11 for all tested concentrations after 48 h. Compared to the control cells (0.1% *v*/*v* DMSO), proliferation was reduced significantly to 60.53% ± 5.32% for 1 µM and up to 34.34% ± 18.27% for 5 µM and 15.91% ± 6.26% for the highest concentration of 10 µM AV25R (Figure 3). Similar results were obtained after 72 h of incubation (Supplementary Materials, Figure S1). The B-ALL cell line SEM showed a slight, but significant, reduction in cell count for the highest concentration to 70.04% ± 5.21%. For the lymphoma cell line SUP-T1, the cell count decreased significantly to 64.65% ± 19.32% (5 µM) and 61.18% ± 17.83% (10 µM), whereas the cell count of SU-DHL-4 just decreased significantly for the highest concentration to 53.08% ± 19.75% compared to the DMSO control.

**Figure 4:**
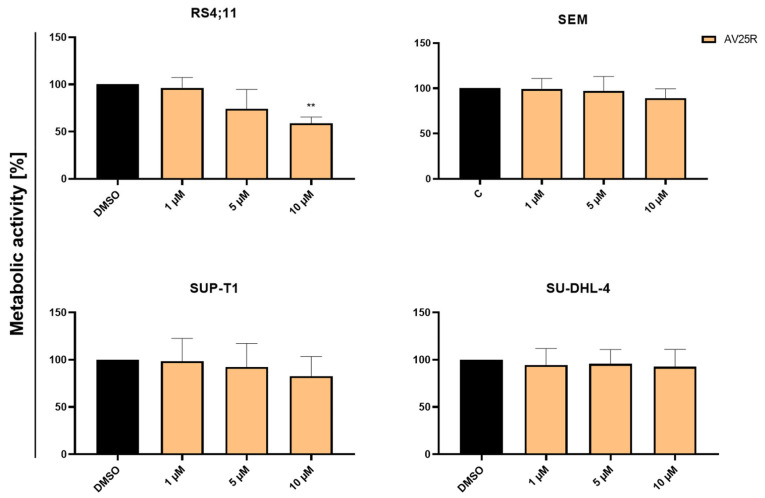
Results of WST-1 after 72 h of concentration-dependent AV25R exposure on B-ALL cell lines RS4;11 and SEM, as well as lymphoma cell lines SUP-T1 and SU-DHL-4. Data are presented as relative values (DMSO control (C) was set as 100%) as mean ± SD (*n* ≥ 3). Significances were displayed as follows: **: *p* < 0.002.

Besides the elicitation of proliferation data, metabolic activity was measured via WST-1 assay after 48 and 72 h, respectively. The B-ALL cell line RS4;11 was the only cell line which responded significantly to the application of thiazolopyridine AV25R at a concentration of 10 µM. Compared to the DMSO control, metabolic activity decreased significantly to 58.83% ± 6.74% (Figure 4). Application of 5 µM AV25R showed reductive tendencies of metabolic activity, which could not be proved as statistically significant. Measurement of metabolic activity of RS4;11 cells after 72 h revealed a significant decrease in metabolic activity to 82.32% ± 4.46% (5 µM) and up to 59.39% ± 6.61% for the highest concentration of 10 µM AV25R (Supplementary Materials, Figure S2). All of the other tested cell lines, including the B-ALL cell line SEM and both lymphoma cell lines SUP-T1 and SU-DHL-4, did not show a reduction in metabolic activity after 48 h and 72 h, respectively.

Based on the results of the cell count after 48 h, we calculated the IC50 values of AV25R for each of the tested cell lines. The highest IC50 was observed for cell line SEM with a value of 20.98 µM. SUP-T1 and SU-DHL-4 showed similar IC50 values of 12.17 µM and 12.72 µM. In comparison, the IC50 value of the RS4;11 cell line is 1.95 µM, which is about six times lower compared to the lymphoma cell lines and ten times lower than the B-ALL cell line SEM (Table 1).

#### 2.1.3. Induction of Apoptosis and Morphological Characterization

Flow-cytometric analysis after Annexin V/FITC and PI double staining was used to reveal a potential pro-apoptotic of thiazolopyridine AV25R. Measurement was carried out after 48 and 72 h of incubation for all used cell lines here.

**Figure 5:**
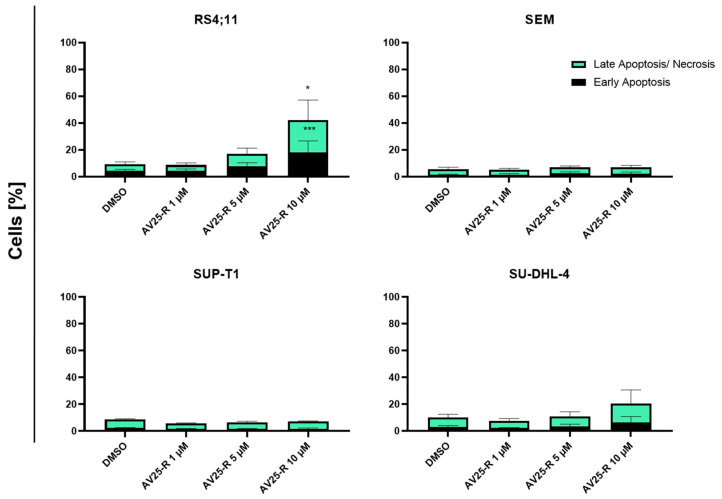
Apoptosis induction in all tested cell lines after concentration-dependent thiazolopyridine exposure. Apoptosis induction was determined by using Annexin-V FITC/PI double staining after an incubation period of 72 h. Shown are means with ± SD (n ≥ 3). Statistical significance was calculated by one-way ANOVA. Significances were displayed as follows: *: *p* < 0.033 and ***: *p* < 0.001.

Except for the B-ALL cell line RS4;11, we could not observe significant induction of apoptosis after 72 h of incubation with AV25R. For the B-cell lymphoma cell line SU-DHL-4, we could observe inductive tendencies for the highest concentration, which was not significant. For the RS4;11 cell line, we identified a significant induction of early and late apoptosis/necrosis for the concentration of 10 µM AV25R. Compared to the DMSO control, 10 µM AV25R led to a significant increase in early apoptotic cells from 4.27% ± 1.30% (control) to 18.27% ± 8.40%. In the case of late apoptotic/necrotic cells, we detected an increase from 4.91% ± 1.60% in the DMSO control up to 23.92% ± 12.93% for the AV25R-treated cells (Figure 5).

**Figure 6:**
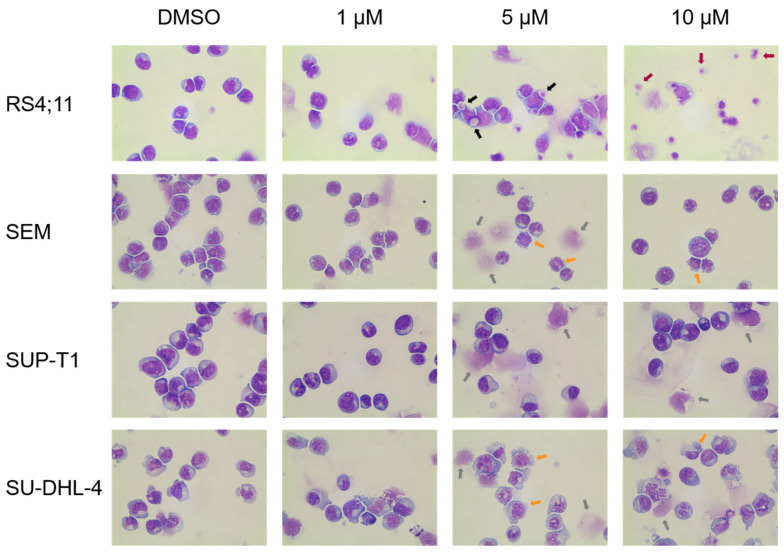
Morphological characterization of RS4;11, SEM, SUP-T1 and SU-DHL-4 cell after exposure to 10 µM AV25R for 72 h. DMSO was used as a control. Black arrows point to cell constriction, red arrows to cell detritus/fragmented cells, orange arrows to membrane blebs and grey arrows to nucleus shadows.

The cell morphology of the four cell lines RS4;11 (B-ALL), SEM (B-ALL), SU-DHL-4 (B cell Lymphoma) and SUP-T1 (T-cell Lymphoma) was investigated by May-Gruenwald-Giemsa (Pappenheim) staining after 72 h exposure to thiazolopyridine AV25R, at a magnification of 100 and in comparison to DMSO control samples (Figure 6). Compared to the DMSO control cells, the studied thiazolopyridine exposure resulted in a clear indication for apoptosis and cellular stress for the B-ALL cell line RS4;11. This is demonstrated by the presence of apoptotic bodies, cellular fragmentation and cell detritus, especially for 5 µM and 10 µM AV25R. Also, the B-ALL cell line showed morphological changes due to AV25R exposure. Here we observed membrane blebs and nucleus shadows, which also indicates an enhanced stress level after application. Morphological analysis of the lymphoma cell lines SUP-T1 and SU-DHL-4 revealed nuclear shadows for both cell lines and the appearance of membrane blebs for the cell line SU-DHL-4. All cell lines show signs of cellular stress compared to the DMSO control. The strongest morphological changes were determined for the B-ALL cell line RS4;11.

#### 2.1.4. Cell Cycle Analysis

**Figure 7:**
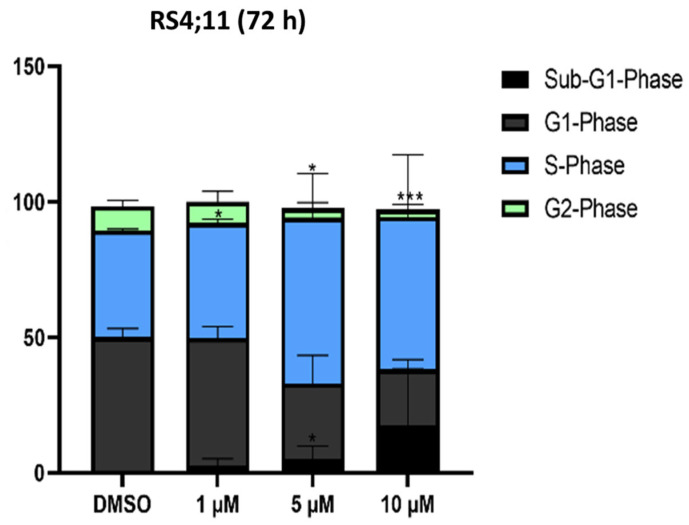
Cell cycle analysis after 72 h thiazolopyridine exposure on RS4;11 cell line. The cell cycle phases are divided into sub-G1, G1, S and G2 phases. Shown is the percentage of cells in the corresponding phase in mean values with standard deviations (*n* = 3). Significances were displayed as follows: *: *p* < 0.033 and ***: *p* < 0.001.

In addition to the flow cytometric apoptosis analysis, we investigated cell cycle analysis after 48 h and 72 h of incubation with thiazolopyridine AV25R, to check if it impacts the cell cycle of our four tested cell lines. After both time points, we did not see any changes in the cell cycle of the B-ALL cell line SEM and lymphoma cell lines SUP-T1 and SU-DHL-4 (Supplementary Materials, Figure S3). After 72 h AV25R exposure, a significant reduction in G1 phase cells was observed for the 5 µM and 10 µM concentrations. When 5 µM AV25R was applied, the proportion was significantly reduced from 50.10% ± 2.97% in the DMSO control to 28.03% ± 10.45% and for 10 µM to 20.75% ± 0.07%. The G2 phase fraction could be decreased from 8.97% ± 2.29% to 2.75% ± 1.82% for 5 µM AV25R. In addition, a small but significant increase in the percentage of cells in S-phase from 39.13% ± 0.33% in the DMSO control to 42.47% ± 1.29% was observed for the concentration of 1 µM AV25R (Figure 7).

### 2.2. Evaluation of Gene Expression and Enrichtment Analysis

#### 2.2.1. Microarray Analysis

**Figure 8:**
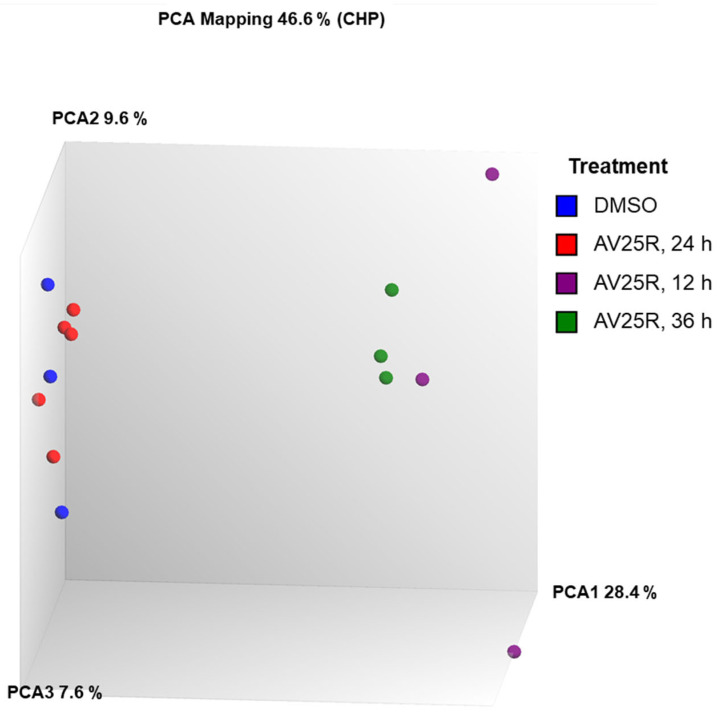
Principal component analysis (PCA) plot of Microarray data of RS4;11 cell line. Blue dots represent DMSO control cells, and red dots represent the results of RS4;11 cells after 24 h of incubation with 5 µM AV25R. The same applies to purple dots (24 h) and green dots (36 h).

To characterize the potential mechanism of action or involved pathways and genes, we employed microarrays for expression profiling from the RS4;11 cell line after exposure to 5 µM AV25R after 12 h, 24 h and 36 h of incubation. For comparison, we also carried out a DMSO control group, to exclude the effects from the used solvent. The results of principal component analysis are shown in Figure 8 and indicate a clear separation of AV25R exposed cells after 12 h (violet) and 36 h (green) compared to the DMSO control (blue). Interestingly, the data set after 24 h (red) shows no distance from the DMSO control group.

**Figure 9:**
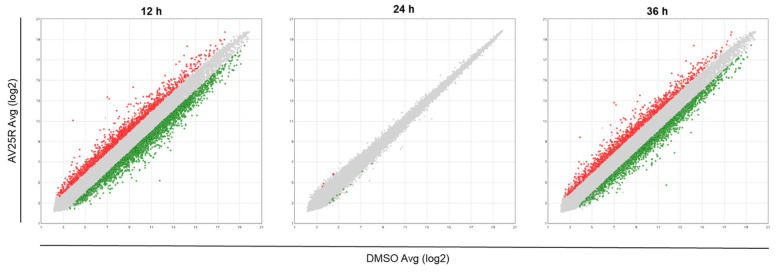
Scatter plots of differentially expressed genes due to AV25R exposure versus DMSO control cells after 12 h (**left**), 24 h (**middle**) and 36 h (**right**). Down-regulated genes are marked in green and up-regulated genes in red. Grey dots represent genes, which did not pass the filter criteria (fold change: >2, <−2, *p*-value < 0.001 (limma).

Overall, we identified 3821 differentially expressed genes after 12 h exposure of RS4;11 with AV25R, including 1384 up-regulated and 2437 down-regulated genes, in total. Results after 36 h exposure show similar results. Here, we identified 2900 DEGs, which passed the filter criteria. Therefrom, 1137 genes were found to be up-regulated and 1763 down-regulated. As the PCA plot already indicated, the data set after 24 h of exposure to AV25R only shows 15 DEGs which passed the filter criteria (Figure 9).

**Figure 10:**
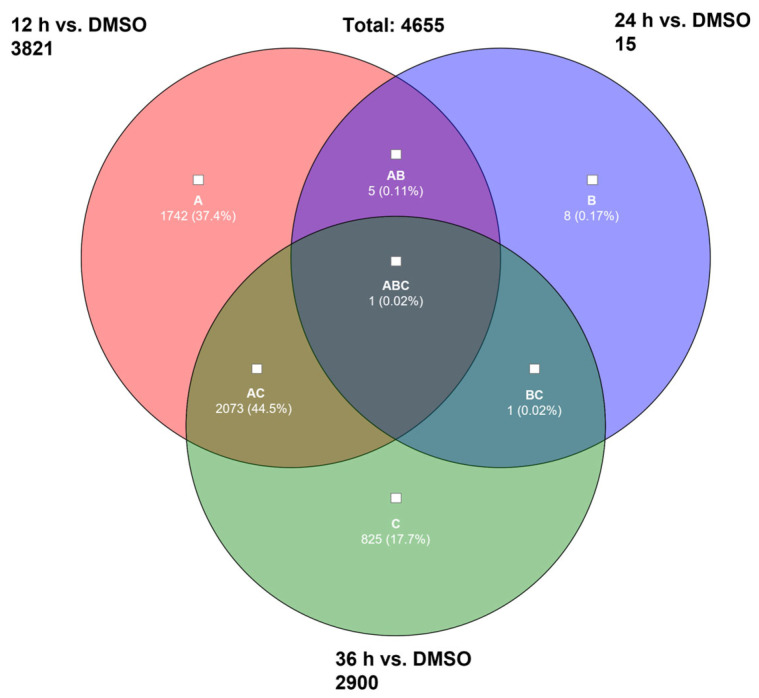
Venn-diagram of overlap of differentially expressed genes due to AV25R exposure versus DMSO control cells after 12 h (A), 24 h (B) and 36 h (C). Filter criteria were set as fold change: >2, <−2, *p*-value < 0.001.

We found 4.655 differentially expressed genes due to AV25R application over all time points. We discovered only one DEG, shared across all tested timepoints (Figure 9, ABC), which accounts for a share of 0.02% of the total amount. A large overlap was observed after 12 h and 36 h of incubation. Here, we identified 2073 shared DEGs, which represent 44.5% of the total DEGs (Figure 10, AC). The highest amount of exclusively differentially regulated genes is 1742 DEGs (A) after 12 h of incubation. Data after 36 h shows 825 exclusively DEGs (C), whereas results after 24 h show 8 DEGs (B).

Integrated with the TAC (Transcriptome Analysis Console software (version 4.0.1), Applied Biosystems/Thermo Fisher Scientific, Waltham, MA, USA), an enrichment analysis tool is provided using the WikiPathways database. In total, we found 1046 differentially regulated pathways after 12 h, 19 pathways after 24 h and 980 pathways after 36 h vs. DMSO control. We extracted the top ten pathways ranked by significance for each timepoint in Table 2. Results after 12 h and 36 h show an overlap of 5 out of 10 pathways, which are addressed after AV25R application. Results after 24 h did not show an overlap with the other time points. After 12 h, the most significant pathway is the “VEGFA-VEGFR2 signaling pathway”, which includes 87 DEGs; after 36 h, it has the same ranking, but with 58 DEGs involved. The second most significant pathway after 12 h represents the “ciliary landscape” with 56 involved DEGs. This pathway also plays an important role after 36 h. Here, it is ranked as the third pathway with 39 DEGs. The “electron transport chain: OXPHOS system in mitochondria” also seems to play a role, as it is ranked as the third for 12 h and 10th after 36 h with a number of 39 and 22 including DEGs.

As the results from the microarray analysis after 24 h indicated, no strong changes, compared to the DMSO control, could be observed either. For all top ten deregulated pathways, there is one DEG assigned to each pathway, and all of them are downregulated after 24 h. This is accompanied by a considerably lower significance level.

#### 2.2.2. Enrichment Analysis

**Figure 11:**
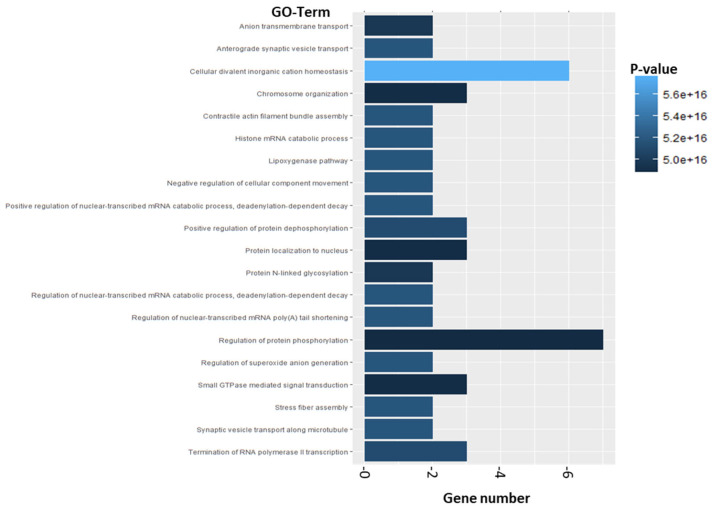
Enrichment analysis from top 500 differentially expressed genes. Analysis is based on results of microarray analysis of RS4;11 cells which were exposed to 5 µM AV25R for 12, 24 and 36 h (compared to DMSO control). Shown are the top 20 GO-Terms of biological processes which were enriched after thiazolopyridine exposure. X-axis represents the gene number of included genes. Color grading is shown according to the dedicated *p*-value.

Enrichment analysis was carried out using the Top 500 differentially expressed Genes. The EnrichR, among others, includes the GO-Term Analysis (Biological Process, Hercules, CA, USA) and KEGG Pathway Analysis. The top 500 DEGs revealed, in total, 1783 significant GO-Terms. The most significant GO-Term is “Cellular covalent inorganic cation homeostasis” with a gene number of 6 DEGs which are involved in this process. The second most significant GO-Term ranked by *p*-value is “Anterograde synaptic vesicle transport” (Figure 11). The DEGs AP3M2 and BLOC1S1 are dedicated to this GO-Term (Supplementary Materials, Table S5).

**Figure 12:**
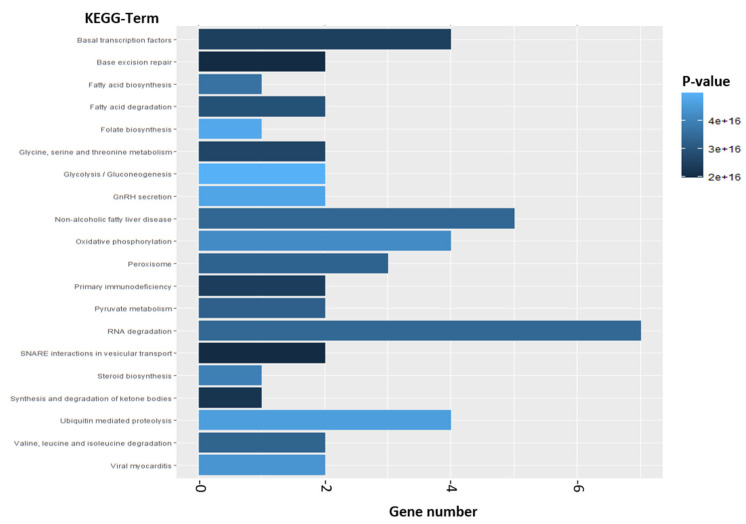
Enrichment analysis from top 500 differentially expressed genes. Analysis is based on results of microarray analysis of RS4;11 cells which were exposed to 5 µM AV25R for 12, 24 and 36 h (compared to DMSO control). Shown are the top 20 KEGG-Terms from KEGG Pathways which were enriched after Thiazolopyridine exposure. X-axis represents the gene number of included genes. Color grading is shown according to the dedicated *p*-value.

In addition to the GO-Term analysis, our enrichment analysis included KEGG Pathway analysis. In total, we found 190 KEGG Pathways which are significantly addressed after exposure of RS4;11 cells to thiazolopyridin AV25R (Supplementary Materials, Table S6). Figure 12 shows the top 20 KEGG-Terms. The three most significant pathways we evaluated (ranked after *p*-value) are “Glycolysis/Gluconeogenesis”, “GnRH secretion” and “Folate Biosynthesis”. The gene number ranges from one to seven genes within these top 20 KEGG Terms (Figure 12).

### 2.3. In Silico Profiling of AV25R

#### 2.3.1. 3D Conformation of AV25R

**Figure 13:**
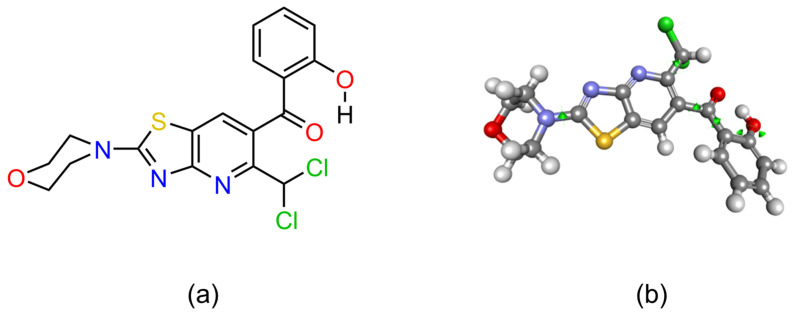
Structure of AV25R. (**a**) 2D molecular structure; and (**b**) 3D conformation of AV25R after minimization. All of the potential rotatable bonds are highlighted with green spindles, which provides flexibility to AV25R to attain various 3D conformations in the receptor binding sites.

The 3D structure of AV25R was prepared from the benzothiazole scaffold (PubChem CID: 7222) using the chemical sketching toolbar of the Biovia Discovery Studio 2022 software suite (DS2022). After adding all of the components, the 3D conformation of AV25R was minimized using the ‘Smart Minimizer’ algorithm for 2000 steps with the CHARMm forcefield in DS2022. The 2D structure and optimized 3D conformation of AV25R are shown in Figure 13.

#### 2.3.2. Computation of ADMET Descriptors for AV25R

**Figure 14:**
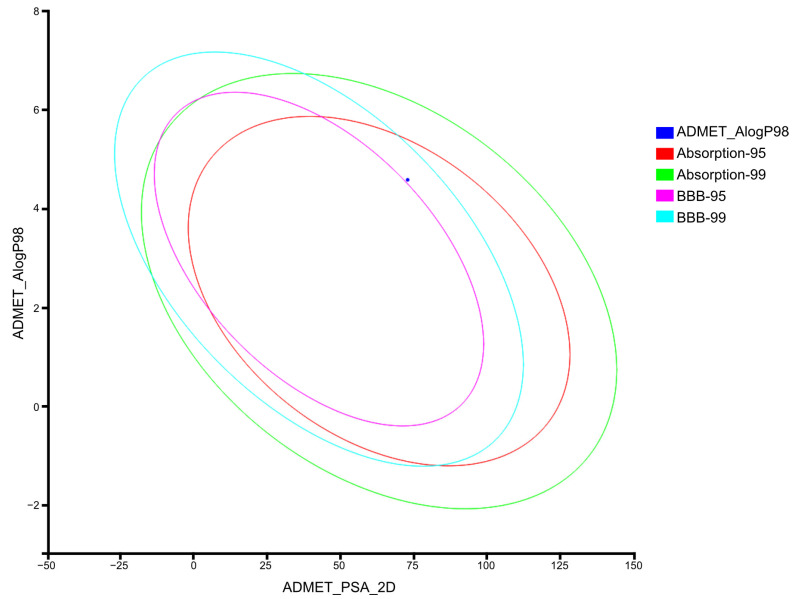
AV25R (blue dot) plotted on PSA-AlogP98 axes indicating that the compound has a very good human intestinal absorption level and high penetrant value for blood-brain barrier after oral administration.

Calculating ADMET descriptors early in the development of a drug is important to avoid eliminating compounds with unfavorable ADMET characteristics later in the development process, preferably before synthesis. As indicated in Figure 14, AV25R has high absorption and penetrating blood-brain barrier capability. The compound AV25R was predicted as a non-inhibitor of the CYP2D6 enzyme, which metabolizes a wide range of substrates in the liver. In general, the compounds inhibiting CYP2D6 constitute, in a majority of cases, drug-drug interactions. The aqueous solubility level of two was predicted for the AV25R, which suggests that the molecule is soluble.

#### 2.3.3. Prediction of Biological Targets of AV25R

In silico profiling for AV25R biological targets was carried out using the ‘Ligand Profiler’ protocol available in the Biovia Discovery Studio 2022 Software suite (DS2022), which maps the molecule to a set of pharmacophores present in the PharmaDB. The PharmaDB database contains over 250,000 pharmacophore models derived from 16,304 entries from the 2017 release of the sc-PDB protein data bank (http://bioinfo-pharma.u-strasbg.fr/scPDB (accessed on 15 July 2023)). A total of 76 unique human proteins were identified to interact with AV25R using pharmacophore screening (Supplementary Materials, Table S1). The top 10 unique targets based on the ‘Fit Value’ for AV25R are shown in Table 3.

We evaluated the binding affinity of AV25R with the experimentally validated ligands in the active site of the proteins from PDB files on which the pharmacophore model was developed using the available ‘CDOCKER’ protocol (DS2022). For each comparison, 10 random conformations of AV25R and experimentally known ligands were generated using high-temperature MD, which was then translated into the active sites of the proteins. Candidate poses were then created using random rigid-body rotations followed by simulated annealing. Afterward, a final minimization was used to refine the ligand poses in the binding cavity.

The top target identified for AV25R is Ferrochelatase (FECH), a terminal enzyme in heme biosynthesis, responsible for catalyzing the insertion of the Fe^2+^ ion into protoporphyrin IX. Molecular docking analysis suggests that AV25R has a very high binding affinity (−CDOCKER Energy: 13.15 kcal/mol) with FECH in comparison to the experimentally validated interactor cholic acid (−CDOCKER Energy: −31.70 kcal/mol; PDB ID: 2HRC). The second-best target identified for AV25R is Methionine aminopeptidase 1 (MAP11) with a binding energy of: 19.37 kcal/mol (−CDOCKER). Our analysis also suggests that AV25R binds to the binding pocket of Glutathione S-transferase P (GSTP1) with a good binding affinity (−CDOCKER Energy: 13.41 kcal/mol). The fourth top target is the Peptidyl-prolyl cis-trans isomerase FKBP1A (FKB1A) protein with a lower binding affinity (−CDOCKER Energy: 12.34 kcal/mol).

Other potential protein targets for AV25R include Serine/threonine-protein kinase Chk2 (CHEK2), Epidermal growth factor receptor (EGFR), Tyrosine-protein kinase JAK2 (JAK2), Protein-tyrosine kinase 2-beta (PTK2B), TGF-beta receptor type-1 (TGFBR1) and E3 ubiquitin-protein ligase Mdm2 (MDM2).

**Figure 15:**
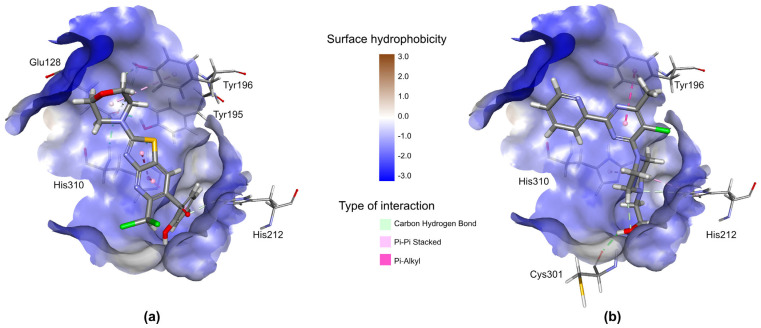
Interaction of (**a**) AV25R and (**b**) Nutlin-3a (a benchmark inhibitor of MDM2) on the p53 binding site of MDM2. It should be noted that AV25R interacts with all the amino acid residues that make bonds with Nutlin-3a except Cys301. In addition, Av25R also interacts with Glu128 and Tyr195 in the same binding cavity of MDM2. The binding cavity of MDM2 is shown as a surface with color in the hydrophobicity index. Both the ligands are shown as a stick model. Interacting amino acid residues are labeled and shown as a stick model.

We compared the binding affinities of AV25R and the previously known benchmark inhibitor of MDM2, Nutlin-3a (PDB ID: 4HG7), using the CDOCKER receptor-ligand docking protocol of DS2022. As shown in Figure 15, AV25R binds to the same p53-binding site of MDM2 with an almost similar binding affinity (−CDOCKER Energy: 12.91 kcal/mol) compared to Nutlin-3a (−CDOCKER Energy: 13.89 kcal/mol).

## 3. Discussion

We evaluated and characterized, for the first time, the biological effects of the thiazolopyridin AV25R [33]. The class of thiazolopyridines were described to have several biological effects including anti-viral [21,22] and anti-microbial [32,34] activity. Further, this molecule class was reported to play a role in the treatment of Parkinson’s disease as well [35].

Herein, the effect of the thiazolopyridine AV25R on cell proliferation and metabolic activity was evaluated in a set of hematological neoplastic cell lines. Interestingly, we saw strong anti-proliferative and metabolism-modulating effects for the B-ALL cell line RS4;11. Application of AV25R at the highest concentration of 10 µM led to a reduction in cell count of up to ~16% compared to DMSO control cells. For the other B-ALL cell line SEM and lymphoma cell lines SUP-T1 and SU-DHL-4, we observed an anti-proliferative effect, which, however, was not as strong as for the RS4;11 cell line. On the other hand, AV25R did not show any significant effects on the metabolic activity of the SEM, SUP-T1 and SU-DHL-4 cell lines, respectively. The same results were confirmed after evaluating the apoptosis-induction due to thiazolopyridine application. In contrast to the SEM cell line, we detected a significant increase in early and late apoptosis for the RS4;11 cell line. Also, slight changes in the cell cycle were detected for RS4;11 exclusively. A possible reason for the non-responding of SEM, even though it is the same entity, could be the genetic background of the cell line’s origin. This is also a major challenge in clinical therapeutic settings, where partially patients show a good response to a certain therapeutic intervention while others do not [36]. These challenges can be addressed partly through the use of precision diagnostics and therapies in oncology, and by using biomarkers for therapy stratification [37]. Chromosomal rearrangements can also play a distinct role for differentially responding to therapeutics and prognosis in cancer therapy [38]. In acute lymphoblastic lymphoma, the chromosomal region 11q23 is often affected by rearrangements [39]. As both B-ALL cell lines RS4;11 [40] and SEM [41] carry this rearrangement at t(4;11), this classifier apparently does not seem to be a primarily relevant factor in the reaction to AV25R exposure. The IC50 value for RS4;11 (1.95 µM) is about ten times lower as that in the cell line SEM (20.98 µM), which indicates another reason for the different response of the two B-ALL cell lines.

Microscopic analysis after exposure to AV25R revealed morphological changes for all four cell lines. For lymphoma cell lines SU-DHL-4 and SUP-T1, as well as the B-ALL cell line SEM, we observed signs of cell stress due to AV25R application. The B-ALL cell line RS4;11 showed strong morphological changes and induction of apoptosis, which could be verified by flow cytometric apoptosis/necrosis assay. Morphological changes due to apoptosis induction include DNA fragmentation, loss of cell shape, formation of membrane blebs, and changes in the cell nucleus [42,43,44].

For an initial assessment of the AV25R potential for an in vivo application, the hemolytic activity of the compound was determined on human whole blood. No hemolytic activity was detected for any of the concentrations used, which is a promising indicator for subsequent in vivo application. These results are consistent with our results from the ADMET analysis, which predicts the absorption, distribution, metabolism, elimination and toxicity (ADMET) properties of new drug candidates. ADMET prediction revealed a high penetrant value for overcoming the human blood–brain barrier after oral uptake. AV25R also appears to show efficient human intestinal absorption potential, which could also be an application method for future in vivo experimental set ups.

Gene expression analyses of RS4;11 cells after incubation with 5 µM AV25R for different timepoints revealed a massive amount of differentially expressed genes, compared to DMSO control cells, after 12 h and 36 h of incubation. Over all timepoints, we identified 4655 DEGs related to the application of thiazolopyridine AV25R. Deregulation after 24 h compared to the DMSO control was far lower compared to other time points. WikiPathway analysis within TAC software also shows the influence at the pathway level, where we observed similar tendencies. After 12 h and 36 h, the “VEGFA-VEGFR2 signaling pathway” is the most significantly differentially regulated pathway. For both time points, there are more downregulated than upregulated genes. The VEGFA-VEGFR2 downstream pathway is related to angiogenesis and vascular permeability, which plays an important role in oncology and represents a potential target signaling pathway for cancer therapy [45]. However, this pathway plays an important role primarily in solid tumor entities, where anti-angiogenic therapies are desirable [46]. Another cancer-related pathway which was addressed after 12 h of AV25R exposure is the EGF/EGFR signaling pathway. The binding of different endogenous ligands to the EGF receptor activates signal cascades, leading to normal cell function. Overexpression of EGFR thus favors tumor growth and progression [47]. Therefore, it represents an interesting target element for cancer therapy and especially targeted cancer therapy.

In silico prediction of molecular targets of AV25R revealed Ferrochelatase (FECH) as the top target. FECH inhibitors have already been shown to possess antiangiogenic properties [48,49]. Molecular docking analysis suggests a very high binding affinity of AV25R to FECH (−CDOCKER Energy: 13.15 kcal/mol), which is even higher than known and experimentally validated FECH interactor cholic acid (−CDOCKER Energy: −31.70 kcal/mol; PDB ID: 2HRC). The second-best target is Methionine aminopeptidase 1 (MAP11), which is known to be over-expressed in several cancer phenotypes, including non-Hodgkin’s lymphoma [50]; pancreatic ductal adenocarcinoma [51]; and acute lymphoblastic leukemia [52]. Interestingly, AV25R has a better binding affinity (−CDOCKER Energy: 19.37 kcal/mol) with MAP11 in comparison to the previously experimentally validated inhibitor PVP (−CDOCKER Energy: 15.85 kcal/mol; PDB ID: 4IKR).

Another identified potential target of AV25R is GSTP1. Its inhibitors have also demonstrated high antiproliferative activities [53], which we identified for thiazolopyridine AV25R. Our analysis suggests that AV25R binds to the same binding pocket of GSTP1 (−CDOCKER Energy: 13.41 kcal/mol) as the previously investigated inhibitor 6-(7-Nitro-2,1,3-benzoxadiazol-4-ylthio) hexanol (−CDOCKER Energy: 20.24 kcal/mol; PDB ID: 3IE3), although with less binding affinity. Interestingly, we have found a downregulation of GSTP1 in the gene expression data after 12 h and 36 h with a fold change of −3.39 (*p*-value: 2.31 × 10^−8^) and −2.5 (3.52 × 10^−6^), respectively (Supplementary Materials, Table S7). This suggests a regulatory relationship between AV25R and GSTP1. AV25R might inhibit signaling pathways by binding to GSTP1, which, in turn, regulate its expression [54]. The downregulation might also be part of a feedback mechanism where GSTP1 protein, or its downstream signaling components, regulate the expression of their own gene in response to substance binding [55].

The fourth top target which we identified through docking analysis is Peptidyl-prolyl cis-trans isomerase FKBP1A (FKB1A). Several previously published studies suggested small molecule inhibitors that interact with the Peptidyl-prolyl cis-trans isomerase FKBP1A (FKB1A) protein and suppress cell proliferation in acute myeloid leukemia phenotypes via inhibition of MTORC1 activity [56,57]. In the microarray data, we observed a slight downregulation of MTOR, which, however, did not pass our strong filtering. The strongest effects were detected after 36 h of incubation with a fold change of −1.68 and a *p*-value of 0.004 (Supplementary Materials, Table S7). It is known that downregulation of MTOR can lead to growth progression, thus representing a possible strategy in cancer therapy [58,59]. Our analysis also indicates that AV25R binds to FKB1A; however, the binding affinity is very low (−CDOCKER Energy: 12.34 kcal/mol) in comparison to other previously known inhibitors of FKB1A (−CDOCKER Energy: 34.28 kcal/mol; PDB ID: 1FKG).

Other potential protein targets for AV25R that are previously described for their role in tumor progression include Serine/threonine-protein kinase Chk2 (CHEK2), Epidermal growth factor receptor (EGFR), Tyrosine-protein kinase JAK2 (JAK2), Protein-tyrosine kinase 2-beta (PTK2B), TGF-beta receptor type-1 (TGFBR1) and E3 ubiquitin-protein ligase Mdm2 (MDM2).

The small molecule inhibitors of many of these proteins showed promising results in regulating tumor-associated phenotypes in several preclinical and clinical studies. For example, the MDM2 inhibitors have previously been shown to exert tumor suppression and the activation of apoptotic pathways by interfering with its interaction with p53 in B-Acute Lymphoblastic Leukemia [60].

## 4. Materials and Methods

### 4.1. Chemical Substances

Thiazolopyridine AV25R has been synthesized in one step by the reaction of dichlorochrome 1 with heterocyclic enamine 2 and was isolated with 70% yield [31].

### 4.2. Cell lines and Cell Culture Methods

The human ALL cell lines SEM, RS4;11 (both B-ALL) and the human lymphoma cell lines SU-DHL-4 (B-cell) and SUP-T1 (T-cell) were purchased from DSMZ (Braunschweig, Germany). The cells were cultivated as recommended by the manufactural protocol. All of the cell lines were cultivated at 37 °C and 5% CO_2_ in the corresponding media with 10–15% heat-inactivated FCS (Biochrom, Berlin, Germany) and 100 µg/mL penicillin and streptomycin (Biochrom, Berlin, Germany).

### 4.3. Drug Exposure Experiments

The suspension cell lines (3.3 × 10^5^ cells) were treated with each substance in three different concentrations (1 µM, 5 µM and 10 µM). Therefore, the cells were cultured in the appropriate medium containing 0.1% (*v*/*v*) DMSO as a control or dose ranges of the different derivatives as a single substance for 24 h, 48 h and 72 h, depending on the experimental assay. After the incubation period, the effect on cell proliferation (trypan blue staining), metabolism (WST-1 assay), apoptosis/necrosis (annexin V/PI staining), cell cycle cell (PI) and morphology were determined. All experiments were performed at least in three biological replicates.

### 4.4. WST-1 Assay

Hematological cell lines SEM, RS4;11, SU-DHL-4 and SUP-T1 were seeded in a 96-well plate at a density of 5 × 10^4^ cells per well in 150 µL media containing the substances or DMSO (0.1% *v*/*v*) as a control. All experiments were carried out in biological and technical replicates. After an incubation period of 48 and 72 h, 15 µL of WST-1 reagent was added to each well and the plates were again incubated for the next 3 h. The absorbance at 450 nm and a reference wavelength at 750 nm were determined using the GloMax-Multi Microplate Multimode Reader (Promega, Madison, WI, USA). For the background control, 150 µL of the appropriate media containing 15 µL WST-1 was used.

### 4.5. Proliferation Assay

To evaluate the impact of newly synthesized thiazolopyridine, cell counts were performed for the hematological cell lines SEM, RS4;11, SU-DHL-4 and SUP-T1. Therefore, 5 × 10^5^ cells were seeded in 24-well plates and exposed to AV25R (1 µM, 5 µM, 10 µM). DMSO (0.1% *v*/*v*) exposed cells served as control. After 48 and 72 h of incubation, the cells were harvested and washed with PBS. The number of viable cells were determined by cell count after trypan blue staining.

### 4.6. Calculation of IC50

To calculate the IC50 values, we used Prism 8 software. Values are calculated based on proliferation results after 48 h of incubation with AV25R. For calculating, we used nonlinear regression → dose–response-inhibition → inhibitor vs. normalized response.

### 4.7. Hemolysis Assay

Hemolytic activity of thiazolopyridine compound was determined by hemoglobin release from whole blood cells. Shortly, whole blood of healthy donors (n = 4) was seeded in 96-well plates (round bottom) and incubated with 1 µM, 5 µM and 10 μM of AV25R for 120 min. Positive control cells (=maximum lysis) were treated with 1% Sodium Dodecyl Sulfate (SDS, Merck KGaA). After an incubation period of 2 h, cell-free supernatants were transferred into a new 96-well plate (flat bottom) and absorption was measured on a GloMax-Multi Microplate Multimode Reader (Promega, Madison, WI, USA) at 540 nm. Hemolytic activity was quantified according to the following formula: % hemolysis = ((OD540 nm sample − OD540 nm buffer)/OD540 nm max − OD540 nm buffer)) × 100.

### 4.8. Morphological Characterization

Morphological changes were determined by May-Gruenwald-Giemsa staining. Therefore, the cells (0.5 × 10^6^) were seeded in a 24-well plate (flat bottom) and incubated with the compound in concentration range of 1 µM to 10 µM for 24 h, 48 h and 72 h. After the incubation period, 0.5 × 10^4^ cells were immobilized to cover slides by using the Cytospin 3 technology centrifuge (Shandon, Frankfurt/Main, Germany). After staining, the morphological changes were analyzed by microscopic analysis with EVOS^®^ XL Core Imaging System (AMG, Washington, DC, USA).

### 4.9. Analysis of Apoptosis

Determination of apoptosis induction was carried out by flow cytometric analysis using a FACS Calibur (BD Biosciences, Heidelberg, Germany). After incubation with AV25R for 72 h in different concentrations (1 µM, 5 µM and 10 µM), cells were double-stained with the fluorescent dyes Annexin V-FITC and Propidiumiodid. During apoptosis, the membrane phospholipids were translocated to the cell surface and Annexin V binds to it. Propidiumiodid is a DNA-intercalator, which binds to cellular nucleic acids of late apoptotic and necrotic cells with no intact cell membrane. The cells were harvested and washed twice with cold PBS (10 min, 180 g, 4°C) and the pellet was resuspended in 100 µL Annexin Binding Buffer. A total of 5 µL Annexin V-FITC was added to cell suspension and incubated for 15 min in the dark (RT). After the incubation period, 400 µL Annexin Binding Buffer was added.

### 4.10. Cell Cycle Analysis

Changes within the rates of cell cycle phases (G0/G1, S and G2/M) after 48 h and 72 h of substance incubation was carried out by flow cytometric analysis using FACS Calibur (BD Bioscience, Heidelberg, Germany). After an RNase digest of ethanol-fixed cells, cells were stained with the DNA-Intercalator Propidiumiodid (PI). Emission signals are proportional to DNA mass. Signal peaks were identified for each phase due to the amount of DNA. Thereby, G0/G1-phase cells have one set of paired chromosomes per cell, G2/M phase cells have two sets of paired chromosomes per cell, prior to cell division and S-phase cells have a variable DNA amount during DNA synthesis.

### 4.11. RNA Isolation for Microarray Analysis

In advance, for microarray analyses in three biological replicates, 4 × 10^6^ cells were first incubated with DMSO or with 5 µM AV25R and cultured for 24 h at 37 °C and 5% CO_2_ in an incubator.

The cells were then washed twice with 2 mL PBS for 10 min at 180× *g* and RT, dissolved in 1 mL PBS, and transferred to a 1.5 mL reaction tube. After centrifugation again for 10 min at 180× *g* and RT, the supernatant was discarded, and the pellet was taken up under the fume hood in 700 µL QIAazol and stored at −80 °C until RNA processing.

The isolation of the RNA was performed according to the instructions for the “miRNeasy Mini Kit” from Qiagen (Hilden, Germany). The concentration was determined spectrophotometrically with the NanoDrop spectral photometer (Peqlab, Radnor, UK).

### 4.12. Microarray Analysis

Gene expression analyses were performed based on minimum three independent biological replicates using Human Clariom D Arrays (Applied Biosystems/Thermo Fisher Scientific, Waltham, MA, USA) according to the manufacturer protocol.

The implementation was outsourced to the Core Facility of Genomics (University Medical Center, Rostock, Germany). Briefly, RNA integrity was verified on a Bioanalyzer 2100 device (Agilent, Santa Clara, CA, USA) and RIN values ≥ 8 were considered as applicable. A quantity of 200 ng total RNA was used for synthesis of first-strand cDNA, followed by second-strand- and cRNA- synthesis. A bead-based method was used for cRNA purification. Further, 215 μg cRNA was applied for second –cycle single-stranded cDNA synthesis, followed by RNaseH hydrolysis, and purification. Ss cDNA was fragmented and labeled afterwards. Hybridization was then performed in the GeneChip Hybridization Oven 645 (Applied Biosystems/Affymetrix, Santa Clara, CA, USA) at 45 °C overnight and washing and staining was carried out using the Fluidics station 450 (Applied Biosystems/Affymetrix, Santa Clara, CA, USA). Microarrays were scanned in the GeneChip Scanner 3000 7G (Applied Biosystems/Affymetrix, Santa Clara, CA, USA) at a resolution of 0.7 µm [61]. TAC software (Applied Biosystems/Thermo Fisher Scientific, Waltham, MA, USA) was used for Microarray data analysis. The probe level analysis was carried out by RMA (robust multichip average normalization) using the signal space transformation correction (SST-RMA) DEGs that passed a threshold of|Fold Change ≥2/≤−2 and a *p*-value < 0.001 (limma) were considered analytically valuable and proceeded to the following enrichment analysis.

### 4.13. Enrichment Analysis

Enrichment analysis was carried out using the top 500 differentially expressed genes of all tested timepoints (12 h, 24 h and 36 h), which passed the stringent filter criteria after TAC software analysis. EnrichR was used for implementation of enrichment analysis. We analyzed GO-Terms (Biological Process), as well as KEGG Pathway analysis, to evaluate the involved pathways.

### 4.14. In Silico Profiling of AV25R

ADMET properties of AV25R were calculated using the ‘ADMET Descriptors’ protocol available in the Biovia Discovery Studio 2022 software suite version 22.1.0.21297 (DS2022). The following ADMET descriptors were included in analysis: (1) aqueous solubility, which predicts the solubility of the compound in water at 25 °C; (2) blood–brain barrier penetration to predict the ability of AV25R to cross the BBB; (3) CYP2D6 binding, to predicts cytochrome P450 2D6 enzyme inhibition by the molecule; (4) hepatotoxicity, to predict the occurrence of dose-dependent human hepatotoxicity; (5) intestinal absorption, to predict human intestinal absorption (HIA) after oral administration of the molecule; and (6) plasma protein binding, to predict the likelihood that the molecule will be highly bound to carrier proteins in the blood. In silico profiling for AV25R biological targets was carried out using the ‘Ligand Profiler’ protocol available in DS2022, which maps the molecule to a set of pharmacophores present in the PharmaDB database, containing over 250,000 pharmacophores models derived from 16,304 entries from the 2017 release of sc-PDB protein data bank (http://bioinfo-pharma.u-strasbg.fr/scPDB, accessed on 15 July 2023). We filtered all the AV25R-mapped pharmacophores that are derived from human proteins only and arranged them by the ‘FitValue’ score, which indicates the goodness-of-fit of the ligand in the target-binding cavity. For the top 10 target proteins based on the FitValue score, the binding affinity of AV25R was compared with the experimentally validated ligands on which the initial pharmacophore models were constructed. For this, the molecular docking analyses were performed using the ‘CDOCKER’ protocol available in the DS2022. For each comparison, the original 3D complex files were downloaded from the PDB database. The receptor molecules were cleaned and corrected for potential errors (e.g., missing loops, alternative conformations, etc.). All of the receptor molecules were mapped with CHARMm36 force field before subjecting to docking analysis. A total of 10 random conformations of AV25R and experimentally known ligands were generated for each of the docking analyses. Each pose is subjected to simulated annealing molecular dynamics followed by the final minimization of the ligand in the right receptor. For each final pose, the CHARMm energy (interaction energy plus ligand strain) and the interaction energy alone was calculated.

### 4.15. Statistical Analysis

All values are constituted as mean ± standard deviation. Prism 8 software was used to determine statistical analysis. After testing for normal contribution, the differences between treated cells and control cells were evaluated using the one-way ANOVA (for normal contributed data) and Kruskal–Wallis (for non-normal contributed data) significances were displayed as follows: *: *p* < 0.033, **: *p* < 0.002, ***: *p* < 0.001 versus the control group.

## 5. Conclusions

In conclusion, our study represents a pioneering effort in evaluating and characterizing the biological effects of the thiazolopyridine AV25R. The thiazolopyridine class, known for its diverse biological activities, has been previously associated with anti-viral, anti-microbial and anti-cancer properties. Our investigation focused on hematological neoplastic cell lines, revealing notable anti-proliferative and metabolism-modulating effects, particularly in the B-ALL cell line RS4;11. While other cell lines demonstrated varying degrees of response, RS4;11 displayed significant morphological changes and apoptosis induction, underscoring the selectivity of AV25R. Moreover, our findings suggest a potential application of AV25R in cancer therapy, as it did not exhibit hemolytic activity in human whole blood, which is a positive indicator for potential in vivo use. ADMET analysis further supported its potential as a drug candidate, predicting favorable absorption, distribution, metabolism, elimination and toxicity properties, including penetration of the blood–brain barrier and intestinal absorption. Gene expression analyses provided insights into the molecular mechanisms underlying AV25R’s effects, revealing a multitude of differentially expressed genes and significant regulation of cancer-related pathways such as VEGFA-VEGFR2 and EGF/EGFR. In silico predictions identified several potential protein targets, with Ferrochelatase (FECH), Methionine aminopeptidase 1 (MAP11) and GSTP1 among the top targets. Molecular docking analyses indicated high binding affinities, particularly with FECH, suggesting anti-angiogenic properties and potential applicability in cancer therapy.

The study concludes that AV25R holds promise as a targeted intervention approach, given its selective impact on cancer cell lines, favorable pharmacological properties, and high affinity for proteins associated with tumor progression. These findings allow further approaches verifying the in silico predicted targets in an experimental set up.

## Figures and Tables

**Table 1:** IC50 values of SEM, RS4;11, SUP-T1 and SU-DHL-4 after incubation with AV25R. Values are calculated based on cell count data after 48 h of incubation.

Cell Line	IC50 Value (µM)
SEM	20.98
RS4;11	1.95
SUP-T1	12.17
SU-DHL-4	12.72

**Table 2:** Results of WikiPathway analysis using TAC software (version 4.0.1.). The top ten differentially regulated pathways are shown from time points 12 h, 24 h and 36 h vs. DMSO control. Pathways are compared across all three timepoints (n.i. means not included at that timepoint).

Pathway	Total Number of DEGs			Up-Regulated DEGs			Down-Regulated DEGs			Significance		
	12 h	24 h	36 h	12 h	24 h	36 h	12 h	24 h	36 h	12 h	24 h	36 h
VEGFA-VEGFR2 signaling	87	n.i.	58	23	n.i.	23	64	n.i.	35	32.71	n.i.	18.73
Ciliary landscape	56	n.i.	39	5	n.i.	6	51	n.i.	33	27.85	n.i.	17.72
Electron transport chain: OXPHOS system in mitochondria	39	n.i.	22	5	n.i.	5	34	n.i.	17	26.12	n.i.	11.68
Nonalcoholic fatty liver disease	44	n.i.	27	7	n.i.	8	37	n.i.	19	23.63	n.i.	12.01
Retinoblastoma gene in cancer	32	n.i.	n.i.	0	n.i.	n.i.	32	n.i.	n.i.	20.74	n.i.	n.i.
miR-targeted genes in lymphocytes	75	n.i.	62	18	n.i.	22	57	n.i.	40	20.32	n.i.	18.36
Proteasome degradation	27	n.i.	n.i.	1	n.i.	n.i.	26	n.i.	n.i.	19.70	n.i.	n.i.
Alzheimer’s disease	51	n.i.	41	10	n.i.	10	41	n.i.	31	18.50	n.i.	15.54
miR-targeted genes in epithelium	57	n.i.	45	13	n.i.	15	44	n.i.	30	17.46	n.i.	14.23
EGF/EGFR signaling pathway	37	n.i.	n.i.	19	n.i.	n.i.	18	n.i.	n.i.	16.55	n.i.	n.i.
Elabela signaling pathway	n.i.	1	n.i.	n.i.	0	n.i.	n.i.	1	n.i.	n.i.	3.15	n.i.
Perturbations to host-cell autophagy, induced by SARS-CoV-2 proteins	n.i.	1	n.i.	n.i.	0	n.i.	n.i.	1	n.i.	n.i.	2.71	n.i.
Steatosis adverse outcome pathway	n.i.	1	n.i.	n.i.	0	n.i.	n.i.	1	n.i.	n.i.	2.58	n.i.
Autophagy	n.i.	1	n.i.	n.i.	0	n.i.	n.i.	1	n.i.	n.i.	2.51	n.i.
JAK-STAT signaling in the regulation of Beta-cells	n.i.	1	n.i.	n.i.	0	n.i.	n.i.	1	n.i.	n.i.	2.45	n.i.
Target of rapamycin signaling	n.i.	1	n.i.	n.i.	0	n.i.	n.i.	1	n.i.	n.i.	2.44	n.i.
Factors and pathways affecting insulin-like growth factor (IGF1)-Akt signaling	n.i.	1	n.i.	n.i.	0	n.i.	n.i.	1	n.i.	n.i.	2.41	n.i.
Neurodegeneration with brain iron accumulation (NBIA) subtypes pathway	n.i.	1	n.i.	n.i.	0	n.i.	n.i.	1	n.i.	n.i.	2.35	n.i.
MTOR signaling	n.i.	1	n.i.	n.i.	0	n.i.	n.i.	1	n.i.	n.i.	2.30	n.i.
DYRK1A involvement regarding cell proliferation in brain development	n.i.	1	n.i.	n.i.	0	n.i.	n.i.	1	n.i.	n.i.	2.19	n.i.
Alzheimer’s disease and miRNA effects	n.i.	n.i.	42	n.i.	n.i.	10	n.i.	n.i.	32	n.i.	n.i.	12.88
RNA pol II transcription—Initiation and elongation	n.i.	n.i.	21	n.i.	n.i.	6	n.i.	n.i.	15	n.i.	n.i.	12.27
miR-targeted genes in muscle cell	n.i.	n.i.	46	n.i.	n.i.	16	n.i.	n.i.	30	n.i.	n.i.	12.08

**Table 3:** Top 10 unique biological targets of AV25R identified from pharmacophore screening of PharmaDB database.

Rank	PDB ID	Target Name	Target Full Name	Target Class	Fit Value
1	2HRC	FECH	Ferrochelatase	Lyase	0.843442
2	4IKR	MAP11	Methionine aminopeptidase 1	Aminopeptidase	0.769355
3	3IE3	GSTP1	Glutathione S-transferase P	Transferase	0.736885
4	1FKG	FKB1A	Peptidyl-prolyl cis-trans isomerase FKBP1A	Isomerase	0.736476
5	3BYZ	DHI1	11-beta-hydroxysteroid dehydrogenase 1	Oxidoreductase	0.702364
6	3ET7	FAK2	Protein-tyrosine kinase 2-beta	Tyrosine-protein kinase	0.649484
7	4NRA	BAZ2B	Bromodomain adjacent to zinc finger domain protein 2B	DNA-binding	0.587567
8	4OJ4	PPARG	Peroxisome proliferator-activated receptor gamma	DNA-binding	0.577672
9	3FZM	HSPA8	Heat shock cognate 71 kDa protein	Chaperone	0.563491
10	3TJS	CYP3A4	Cytochrome P450 3A4	Oxidoreductase	0.482263

## Data Availability

Data are contained within the article or Supplementary Materials.

## Supplementary Materials

The following supporting information can be downloaded at: https://www.mdpi.com/article/10.3390/molecules28248120/s1, Figure S1: Results of cell count after 72 h; Figure S2: Results of WST-1 after 48 h; Figure S3: Cell cycle analysis after 72 h; Table S1: Unique human protein targets of AV25R identified using pharmacophore screening; Table S2: WikiPathway Analysis: 12 h vs. DMSO control.; Table S3: WikiPathway Analysis: 24 h vs. DMSO control.; Table S4: WikiPathway Analysis: 36 h vs. DMSO control.; Table S5: GO-Term Analysis of top 500 deregulated genes.; Table S6: KEGG-Term Analysis of top 500 deregulated genes; Table S7: Comparison of target prediction and gene expression values from Microarray data analysis.
