# Supplementary material for: Exploring Thiazolopyridine AV25R: Unraveling of Biological Activities, Selective Anti-Cancer Properties and In Silico Target and Binding Prediction in Hematological Neoplasms
Source: Molecules. 2023 Dec 15;28(24):8120. doi: 10.3390/molecules28248120 (PMC10745743; doi:10.3390/molecules28248120)
Supplement: Supplementary file 1 [file molecules-28-08120-s001.zip › Supplementary Material/Supplementary Figures.pdf]

Supplementary Materials: Figures

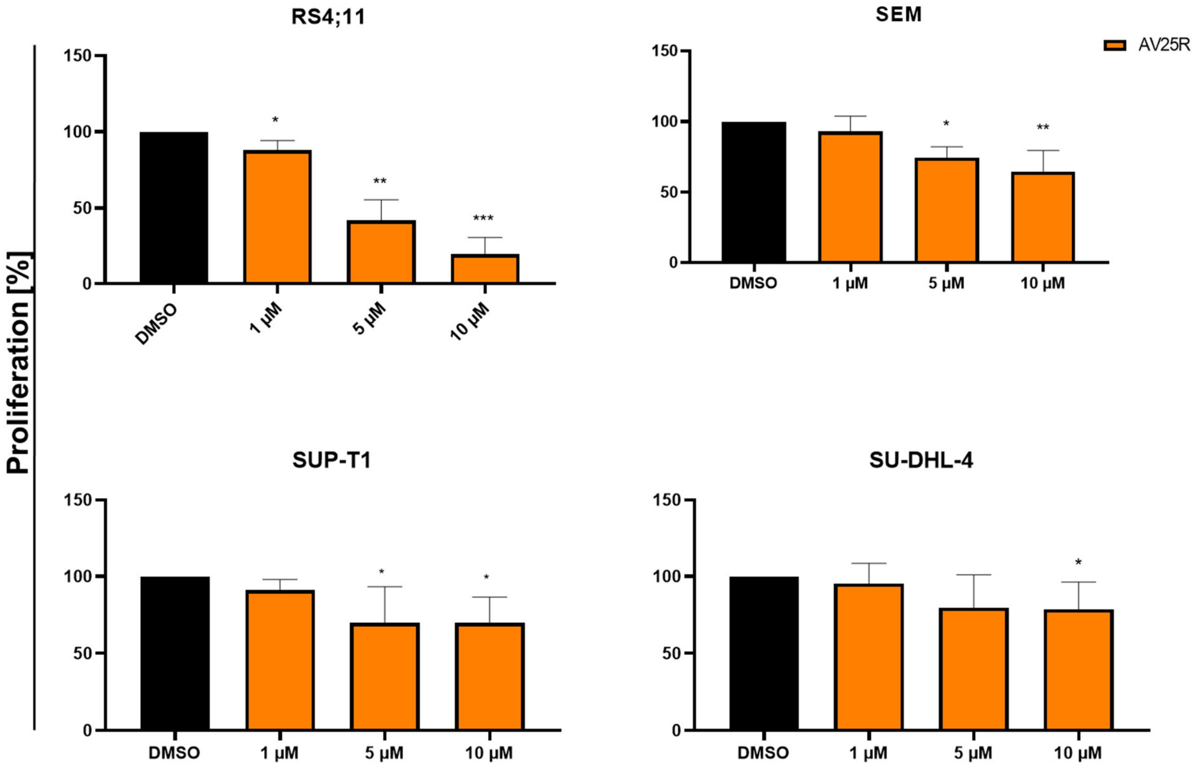

Figure S1: Results of cell count after 72 h of concentration-dependent AV25R exposure on B-ALL cell lines RS4;11 and SEM, as well as lymphoma cell lines SUP-T1 and SU-DHL-4. Data are presented as relative values (DMSO control was set as 100 %) as mean ± SD (n≥3).

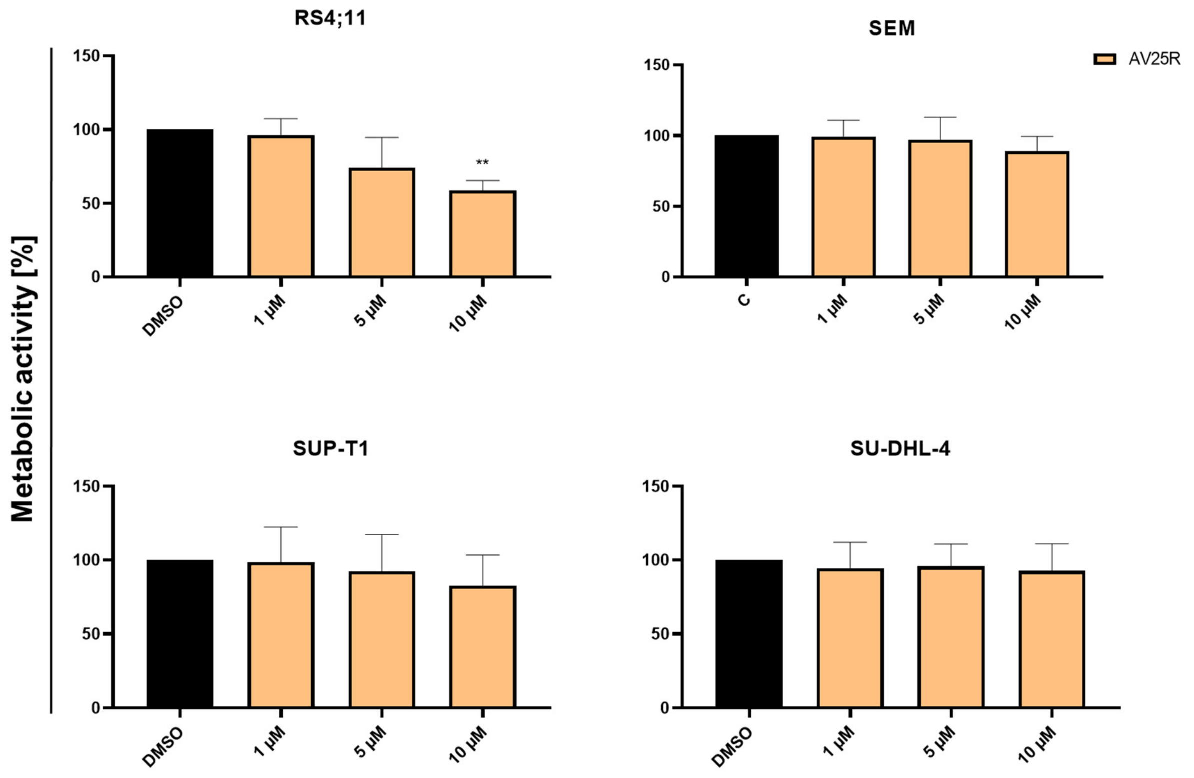

Figure S2: Results of WST-1 after 48 h of concentration-dependent AV25R exposure on B-ALL cell lines RS4;11 and SEM, as well as lymphoma cell lines SUP-T1 and SU-DHL-4. Data are presented as relative values (DMSO control was set as 100%) as mean ± SD (n≥3).

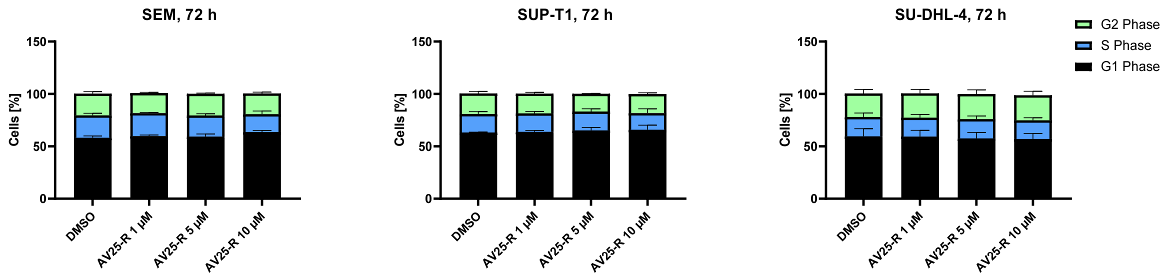

Figure S3: Cell cycle analysis after 72 h thiazolopyridine exposure on SEM, SUP-T1 and SU-DHL-4 cell line. The cell cycle phases are divided into sub-G1, G1, S and G2 phases. Shown is the percentage of cells in the corresponding phase in mean values with standard deviations (n = 3).
